# 1-(5,5-Dioxido-10*H*-phenothia­zin-10-yl)ethanone

**DOI:** 10.1107/S1600536811021854

**Published:** 2011-06-18

**Authors:** M. S. Siddegowda, Jerry P. Jasinski, James A. Golen, H. S. Yathirajan

**Affiliations:** a Department of Studies in Chemistry, University of Mysore, Manasagangotri, Mysore 570 006, India; b Department of Chemistry, Keene State College, 229 Main Street, Keene, NH 03435-2001, USA

## Abstract

In the title compound, C_14_H_11_NO_3_S, the six-membered thia­zine ring fused to two benzene rings adopts a distorted boat conformation. The dihedral angle between the mean planes of the two benzene rings is 45.8 (1)°. The crystal packing is stabilized by weak inter­molecular C—H⋯O inter­actions.

## Related literature

For synthetic dyes and electroluminescent materials containing phenothia­zine, see: Miller *et al.* (1999 ▶). For anti­psychotic drugs, see: Wermuth *et al.* (2003 ▶). For applications of phenothia­zine derivatives in medicine, see: Wang *et al.* (2008 ▶). For their anti­tumor activity, see: Lam *et al.* (2001 ▶). For related structures, see: Harrison *et al.* (2007 ▶); Jasinski *et al.* (2011 ▶). For standard bond lengths, see Allen *et al.* (1987 ▶). For puckering parameters, see: Cremer & Pople (1975 ▶).
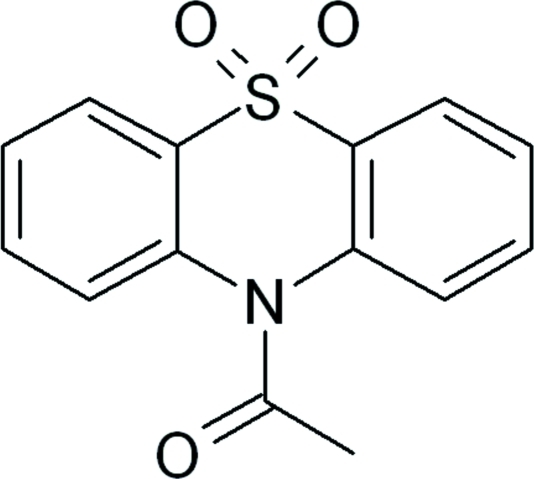


## Experimental

### 

#### Crystal data

C_14_H_11_NO_3_S
*M* *_r_* = 273.30
Monoclinic, 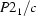

*a* = 12.5715 (6) Å
*b* = 8.7648 (4) Å
*c* = 11.5828 (5) Å
β = 92.142 (4)°
*V* = 1275.38 (10) Å^3^
*Z* = 4
Mo *K*α radiation
μ = 0.26 mm^−1^
*T* = 173 K
0.35 × 0.15 × 0.15 mm

#### Data collection

Oxford Diffraction Xcalibur Eos Gemini diffractometer
Absorption correction: multi-scan (*CrysAlis RED*; Oxford Diffraction, 2010 ▶) *T*_min_ = 0.916, *T*_max_ = 0.963
5342 measured reflections
2597 independent reflections
2263 reflections with *I* > 2σ(*I*)
*R*_int_ = 0.019

#### Refinement

*R*[*F* ^2^ > 2σ(*F* ^2^)] = 0.036
*wR*(*F* ^2^) = 0.103
*S* = 1.02
2597 reflections
173 parameters
H-atom parameters constrained
Δρ_max_ = 0.26 e Å^−3^
Δρ_min_ = −0.40 e Å^−3^

### 

Data collection: *CrysAlis PRO* (Oxford Diffraction, 2010 ▶); cell refinement: *CrysAlis PRO*; data reduction: *CrysAlis RED*; program(s) used to solve structure: *SHELXS97* (Sheldrick, 2008 ▶); program(s) used to refine structure: *SHELXL97* (Sheldrick, 2008 ▶); molecular graphics: *SHELXTL* (Sheldrick, 2008 ▶); software used to prepare material for publication: *SHELXTL*.

## Supplementary Material

Crystal structure: contains datablock(s) I. DOI: 10.1107/S1600536811021854/yk2011sup1.cif

Structure factors: contains datablock(s) I. DOI: 10.1107/S1600536811021854/yk2011Isup2.hkl

Supplementary material file. DOI: 10.1107/S1600536811021854/yk2011Isup3.cml

Additional supplementary materials:  crystallographic information; 3D view; checkCIF report

## Figures and Tables

**Table 1:** Hydrogen-bond geometry (Å, °)

*D*—H⋯*A*	*D*—H	H⋯*A*	*D*⋯*A*	*D*—H⋯*A*
C2—H2*B*⋯O2^i^	0.95	2.50	3.246 (2)	135
C8—H8*A*⋯O1^ii^	0.95	2.54	3.376 (2)	147
C9—H9*A*⋯O3^ii^	0.95	2.56	3.322 (2)	137

Symmetry codes: (i) 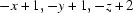; (ii) 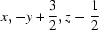.

**Fig. 1.**
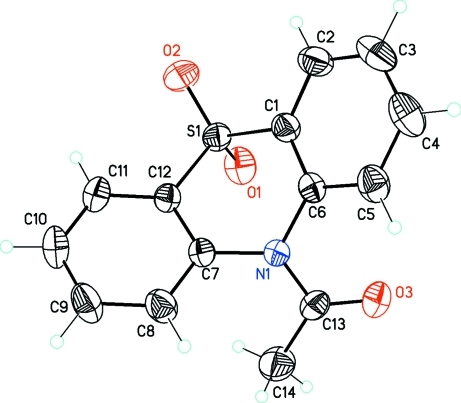
Molecular structure of the title compound showing the atom labeling scheme and 50% probability displacement ellipsoids.

**Fig. 2.**
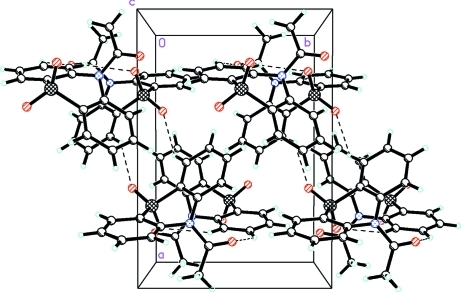
Packing diagram of the title compound viewed down the c axis. Dashed lines indicate weak C—H···O intermolecular interactions.

## supplementary crystallographic information

### Comment

Phenothiazine is a well known heterocycle. The phenothiazine structure occurs in many synthetic dyes, electroluminescent materials (Miller *et al.*, 1999) and drugs, especially various antipsychotic drugs, e.g. chlorpromazine and antihistaminic drugs, e.g. promethazine (Wermuth, 2003). Recently, researchers have found some new applications for phenothiazine derivatives in medicine related to antitubercular (Wang *et al.*, 2008) and antitumor activities (Lam *et al.*, 2001).  The title compound has been used in the synthesis of oxomemazine, an antihistamine and anticholinergic drug of the phenothiazine chemical class used for the treatment of coughs. The crystal structures of dioxopromethazinium picrate (Harrison *et al.*, 2007) and 1-(10H-phenothiazin-2-yl)ethanone (Jasinski *et al.*, 2011) have been reported.  In view of the importance of phenothiazines, this paper reports the crystal structure of the title compound, C_14_H_11_NO_3_S.

In the title compound the six-membered thiazine ring fused to two benzene rings adopts a distorted boat conformation. (Cremer & Pople, 1975) with puckering parameters Q, θ and φ of 0.6257 (12) Å, 95.85 (13)° and 180.29 (15)°, respectively (Fig. 1).  For an ideal boat θ and φ have values of 90.0° and 180°.  The dihedral angle between the mean planes of the two benzene rings is 45.8 (1)°. The ethanone group extends away from corner of the boat crease with a -169.76 (14)° (C6/N1/C13/C14) torsion angle. The SO_2_ group extends away from the opposite corner of the boat crease with a 105.8 (14)° (C2/C1/S1/O1) torsion angle.  Bond lengths are in normal ranges (Allen*et al.*, 1987). Crystal packing is stabiized by weak C—H···O (Table 1, Fig. 2) intermolecular interactions.

### Experimental

The title compound was obtained as a gift sample from RL Fine Chem, Bangalore, India.  X-ray quality crystals were obtained by slow evaporation of solution of a 1:1 mixture of acetone:ethanol (m.p.: 495 K).

### Refinement

All of the H atoms were placed in their calculated positions and then refined using the riding model with Atom—H lengths of 0.95Å (CH), or 0.98Å (CH_3_).  Isotropic displacement parameters for these atoms were set to 1.19-1.21 (CH) or 1.49 (CH_3_) times *U*_eq_ of the parent atom.

### Crystal data

**Table:**

C_14_H_11_NO_3_S	*F*(000) = 568
*M**_r_* = 273.30	*D*_x_ = 1.423 Mg m^−^^3^
Monoclinic, *P*2_1_/*c*	Mo *K*α radiation, λ = 0.71073 Å
Hall symbol: -P 2ybc	Cell parameters from 3700 reflections
*a* = 12.5715 (6) Å	θ = 3.3–32.3°
*b* = 8.7648 (4) Å	µ = 0.26 mm^−^^1^
*c* = 11.5828 (5) Å	*T* = 173 K
β = 92.142 (4)°	Block, colorless
*V* = 1275.38 (10) Å^3^	0.35 × 0.15 × 0.15 mm
*Z* = 4	

### Data collection

**Table:**

Oxford Diffraction Xcalibur Eos Gemini diffractometer	2597 independent reflections
Radiation source: Enhance (Mo) X-ray Source	2263 reflections with *I* > 2σ(*I*)
graphite	*R*_int_ = 0.019
Detector resolution: 16.1500 pixels mm^-1^	θ_max_ = 26.4°, θ_min_ = 3.3°
ω scans	*h* = −15→15
Absorption correction: multi-scan (*CrysAlis RED*; Oxford Diffraction, 2010)	*k* = −10→10
*T*_min_ = 0.916, *T*_max_ = 0.963	*l* = −14→13
5342 measured reflections	

### Refinement

**Table:**

Refinement on *F*^2^	Primary atom site location: structure-invariant direct methods
Least-squares matrix: full	Secondary atom site location: difference Fourier map
*R*[*F*^2^ > 2σ(*F*^2^)] = 0.036	Hydrogen site location: inferred from neighbouring sites
*wR*(*F*^2^) = 0.103	H-atom parameters constrained
*S* = 1.02	*w* = 1/[σ^2^(*F*_o_^2^) + (0.0596*P*)^2^ + 0.3216*P*] where *P* = (*F*_o_^2^ + 2*F*_c_^2^)/3
2597 reflections	(Δ/σ)_max_ = 0.001
173 parameters	Δρ_max_ = 0.26 e Å^−^^3^
0 restraints	Δρ_min_ = −0.40 e Å^−^^3^

### Special details

**Table:**

Geometry. All esds (except the esd in the dihedral angle between two l.s. planes) are estimated using the full covariance matrix. The cell esds are taken into account individually in the estimation of esds in distances, angles and torsion angles; correlations between esds in cell parameters are only used when they are defined by crystal symmetry. An approximate (isotropic) treatment of cell esds is used for estimating esds involving l.s. planes.
Refinement. Refinement of *F*^2^ against ALL reflections. The weighted *R*-factor *wR* and goodness of fit *S* are based on *F*^2^, conventional *R*-factors *R* are based on *F*, with *F* set to zero for negative *F*^2^. The threshold expression of *F*^2^ > σ(*F*^2^) is used only for calculating *R*-factors(gt) *etc*. and is not relevant to the choice of reflections for refinement. *R*-factors based on *F*^2^ are statistically about twice as large as those based on *F*, and *R*- factors based on ALL data will be even larger.

### Fractional atomic coordinates and isotropic or equivalent isotropic displacement parameters (Å^2^)

**Table:**

	*x*	*y*	*z*	*U*_iso_*/*U*_eq_	
S1	0.27860 (3)	0.53431 (4)	0.88913 (3)	0.03458 (15)	
O1	0.18673 (10)	0.57759 (15)	0.95043 (10)	0.0481 (3)	
O2	0.34587 (11)	0.41839 (15)	0.93886 (12)	0.0552 (4)	
O3	0.14803 (10)	0.98466 (14)	0.75069 (12)	0.0492 (3)	
N1	0.22033 (10)	0.75636 (14)	0.71011 (11)	0.0301 (3)	
C1	0.35385 (12)	0.69784 (18)	0.86054 (13)	0.0330 (3)	
C2	0.44865 (13)	0.7260 (2)	0.92270 (15)	0.0441 (4)	
H2B	0.4745	0.6564	0.9801	0.053*	
C3	0.50395 (14)	0.8569 (2)	0.89904 (17)	0.0521 (5)	
H3A	0.5685	0.8793	0.9410	0.063*	
C4	0.46595 (15)	0.9559 (2)	0.81450 (18)	0.0523 (5)	
H4A	0.5045	1.0467	0.7999	0.063*	
C5	0.37288 (14)	0.9262 (2)	0.75021 (15)	0.0415 (4)	
H5A	0.3487	0.9941	0.6909	0.050*	
C6	0.31596 (11)	0.79526 (17)	0.77439 (13)	0.0307 (3)	
C7	0.21083 (11)	0.60193 (17)	0.67173 (13)	0.0303 (3)	
C8	0.18008 (14)	0.5650 (2)	0.55873 (15)	0.0413 (4)	
H8A	0.1651	0.6434	0.5038	0.050*	
C9	0.17142 (16)	0.4131 (2)	0.52666 (16)	0.0486 (5)	
H9A	0.1488	0.3881	0.4498	0.058*	
C10	0.19506 (15)	0.2974 (2)	0.60424 (16)	0.0470 (4)	
H10A	0.1866	0.1939	0.5814	0.056*	
C11	0.23104 (13)	0.33228 (18)	0.71510 (15)	0.0388 (4)	
H11A	0.2510	0.2536	0.7680	0.047*	
C12	0.23754 (12)	0.48386 (17)	0.74801 (13)	0.0301 (3)	
C13	0.13555 (12)	0.85896 (18)	0.70914 (14)	0.0355 (4)	
C14	0.03011 (14)	0.8073 (2)	0.65925 (19)	0.0527 (5)	
H14A	−0.0254	0.8789	0.6816	0.079*	
H14B	0.0140	0.7054	0.6886	0.079*	
H14C	0.0326	0.8039	0.5748	0.079*	

### Atomic displacement parameters (Å^2^)

**Table:**

	*U*^11^	*U*^22^	*U*^33^	*U*^12^	*U*^13^	*U*^23^
S1	0.0430 (2)	0.0320 (2)	0.0286 (2)	0.00086 (16)	−0.00171 (16)	0.00387 (15)
O1	0.0580 (8)	0.0502 (7)	0.0371 (6)	−0.0057 (6)	0.0143 (6)	−0.0048 (6)
O2	0.0673 (9)	0.0431 (7)	0.0538 (8)	0.0057 (6)	−0.0169 (7)	0.0151 (6)
O3	0.0556 (8)	0.0313 (6)	0.0604 (8)	0.0070 (5)	−0.0022 (6)	−0.0060 (6)
N1	0.0334 (6)	0.0253 (6)	0.0311 (6)	−0.0007 (5)	−0.0050 (5)	0.0007 (5)
C1	0.0332 (7)	0.0339 (8)	0.0319 (8)	0.0001 (6)	−0.0005 (6)	−0.0033 (6)
C2	0.0391 (8)	0.0518 (10)	0.0406 (9)	0.0034 (8)	−0.0086 (7)	−0.0069 (8)
C3	0.0363 (9)	0.0687 (13)	0.0509 (11)	−0.0097 (9)	−0.0056 (8)	−0.0147 (10)
C4	0.0475 (10)	0.0565 (12)	0.0532 (11)	−0.0216 (9)	0.0068 (9)	−0.0083 (9)
C5	0.0473 (9)	0.0389 (9)	0.0385 (9)	−0.0100 (7)	0.0036 (7)	0.0000 (7)
C6	0.0322 (7)	0.0300 (7)	0.0299 (7)	−0.0021 (6)	0.0011 (6)	−0.0044 (6)
C7	0.0310 (7)	0.0268 (7)	0.0330 (8)	−0.0024 (6)	−0.0006 (6)	−0.0009 (6)
C8	0.0534 (10)	0.0380 (9)	0.0321 (8)	−0.0081 (8)	−0.0040 (7)	0.0014 (7)
C9	0.0667 (12)	0.0443 (10)	0.0346 (9)	−0.0121 (9)	−0.0004 (8)	−0.0097 (8)
C10	0.0620 (11)	0.0310 (9)	0.0485 (10)	−0.0071 (8)	0.0104 (9)	−0.0115 (8)
C11	0.0467 (9)	0.0269 (8)	0.0432 (9)	0.0013 (7)	0.0080 (7)	−0.0001 (7)
C12	0.0326 (7)	0.0280 (7)	0.0297 (7)	0.0006 (6)	0.0031 (6)	−0.0008 (6)
C13	0.0401 (8)	0.0311 (8)	0.0351 (8)	0.0023 (6)	0.0018 (6)	0.0060 (7)
C14	0.0363 (9)	0.0519 (11)	0.0697 (13)	0.0035 (8)	−0.0019 (9)	0.0010 (10)

### Geometric parameters (Å, °)

**Table:**

S1—O2	1.4292 (13)	C5—C6	1.387 (2)
S1—O1	1.4294 (13)	C5—H5A	0.9500
S1—C12	1.7524 (16)	C7—C8	1.389 (2)
S1—C1	1.7557 (16)	C7—C12	1.394 (2)
O3—C13	1.210 (2)	C8—C9	1.386 (2)
N1—C13	1.394 (2)	C8—H8A	0.9500
N1—C7	1.4283 (19)	C9—C10	1.380 (3)
N1—C6	1.4314 (19)	C9—H9A	0.9500
C1—C6	1.384 (2)	C10—C11	1.380 (2)
C1—C2	1.391 (2)	C10—H10A	0.9500
C2—C3	1.374 (3)	C11—C12	1.384 (2)
C2—H2B	0.9500	C11—H11A	0.9500
C3—C4	1.380 (3)	C13—C14	1.496 (2)
C3—H3A	0.9500	C14—H14A	0.9800
C4—C5	1.388 (3)	C14—H14B	0.9800
C4—H4A	0.9500	C14—H14C	0.9800
			
O2—S1—O1	117.73 (8)	C8—C7—C12	118.45 (14)
O2—S1—C12	110.23 (8)	C8—C7—N1	122.08 (14)
O1—S1—C12	108.36 (7)	C12—C7—N1	119.41 (13)
O2—S1—C1	109.97 (8)	C9—C8—C7	119.52 (16)
O1—S1—C1	109.16 (7)	C9—C8—H8A	120.2
C12—S1—C1	99.91 (7)	C7—C8—H8A	120.2
C13—N1—C7	123.62 (12)	C10—C9—C8	121.26 (16)
C13—N1—C6	118.52 (13)	C10—C9—H9A	119.4
C7—N1—C6	116.52 (12)	C8—C9—H9A	119.4
C6—C1—C2	121.92 (15)	C11—C10—C9	119.88 (16)
C6—C1—S1	117.79 (11)	C11—C10—H10A	120.1
C2—C1—S1	120.29 (13)	C9—C10—H10A	120.1
C3—C2—C1	118.35 (17)	C10—C11—C12	118.89 (16)
C3—C2—H2B	120.8	C10—C11—H11A	120.6
C1—C2—H2B	120.8	C12—C11—H11A	120.6
C2—C3—C4	120.13 (17)	C11—C12—C7	121.87 (15)
C2—C3—H3A	119.9	C11—C12—S1	120.77 (12)
C4—C3—H3A	119.9	C7—C12—S1	117.36 (11)
C3—C4—C5	121.69 (17)	O3—C13—N1	119.75 (15)
C3—C4—H4A	119.2	O3—C13—C14	121.93 (15)
C5—C4—H4A	119.2	N1—C13—C14	118.29 (14)
C6—C5—C4	118.54 (17)	C13—C14—H14A	109.5
C6—C5—H5A	120.7	C13—C14—H14B	109.5
C4—C5—H5A	120.7	H14A—C14—H14B	109.5
C1—C6—C5	119.33 (14)	C13—C14—H14C	109.5
C1—C6—N1	119.19 (13)	H14A—C14—H14C	109.5
C5—C6—N1	121.45 (14)	H14B—C14—H14C	109.5
			
O2—S1—C1—C6	154.78 (12)	C13—N1—C7—C12	−121.42 (16)
O1—S1—C1—C6	−74.65 (14)	C6—N1—C7—C12	45.13 (19)
C12—S1—C1—C6	38.88 (13)	C12—C7—C8—C9	3.4 (2)
O2—S1—C1—C2	−24.71 (16)	N1—C7—C8—C9	−179.49 (16)
O1—S1—C1—C2	105.85 (14)	C7—C8—C9—C10	−1.5 (3)
C12—S1—C1—C2	−140.62 (14)	C8—C9—C10—C11	−1.9 (3)
C6—C1—C2—C3	1.8 (3)	C9—C10—C11—C12	3.3 (3)
S1—C1—C2—C3	−178.75 (14)	C10—C11—C12—C7	−1.3 (2)
C1—C2—C3—C4	−0.8 (3)	C10—C11—C12—S1	177.93 (13)
C2—C3—C4—C5	−1.0 (3)	C8—C7—C12—C11	−2.0 (2)
C3—C4—C5—C6	1.7 (3)	N1—C7—C12—C11	−179.24 (14)
C2—C1—C6—C5	−1.1 (2)	C8—C7—C12—S1	178.73 (12)
S1—C1—C6—C5	179.43 (12)	N1—C7—C12—S1	1.50 (18)
C2—C1—C6—N1	177.23 (14)	O2—S1—C12—C11	26.68 (16)
S1—C1—C6—N1	−2.26 (19)	O1—S1—C12—C11	−103.47 (14)
C4—C5—C6—C1	−0.6 (2)	C1—S1—C12—C11	142.39 (13)
C4—C5—C6—N1	−178.90 (15)	O2—S1—C12—C7	−154.06 (12)
C13—N1—C6—C1	122.59 (15)	O1—S1—C12—C7	75.79 (13)
C7—N1—C6—C1	−44.68 (19)	C1—S1—C12—C7	−38.35 (13)
C13—N1—C6—C5	−59.1 (2)	C7—N1—C13—O3	174.72 (15)
C7—N1—C6—C5	133.59 (15)	C6—N1—C13—O3	8.4 (2)
C13—N1—C7—C8	61.5 (2)	C7—N1—C13—C14	−3.5 (2)
C6—N1—C7—C8	−131.99 (15)	C6—N1—C13—C14	−169.76 (14)

### Hydrogen-bond geometry (Å, °)

**Table:**

*D*—H···*A*	*D*—H	H···*A*	*D*···*A*	*D*—H···*A*
C2—H2B···O2^i^	0.95	2.50	3.246 (2)	135
C8—H8A···O1^ii^	0.95	2.54	3.376 (2)	147
C9—H9A···O3^ii^	0.95	2.56	3.322 (2)	137

Symmetry codes: (i) −*x*+1, −*y*+1, −*z*+2; (ii) *x*, −*y*+3/2, *z*−1/2.
